# ILAE dietary treatments task force special report: Maternal ketogenic diet exposure in epilepsy pregnancy registries—A call to action

**DOI:** 10.1002/epi4.70246

**Published:** 2026-03-25

**Authors:** Magnhild Kverneland, Valentina De Giorgis, Laura Healy, Kelly Faltersack, Tanya J. W. McDonald, Marisa Armeno, Mackenzie C. Cervenka, Anita Devlin

**Affiliations:** ^1^ National Center for Epilepsy, Full Member of European Reference Network EpiCARE Oslo University Hospital Oslo Norway; ^2^ Department of Brain and Behaviour Neuroscience University of Pavia Pavia Italy; ^3^ Child Neurology and Psychiatry Unit, IRCCS Mondino Foundation, Full Member of European Reference Network EpiCARE Pavia Italy; ^4^ Department of Clinical Nutrition St James Hospital Dublin Ireland; ^5^ Department of Clinical Nutrition University of Wisconsin Hospitals and Clinics Madison Wisconsin USA; ^6^ Department of Neurology Johns Hopkins School of Medicine Baltimore Maryland USA; ^7^ Department of Clinical Nutrition Metabolic Research Center Buenos Aires Argentina; ^8^ Department of Paediatric Neurology, Great North Children's Hospital, Newcastle‐Upon‐Tyne & Translational and Clinical Research Institute Newcastle University, Chair of the ILAE Dietary Task Force Newcastle upon Tyne UK

**Keywords:** Glut1 deficiency syndrome, ketogenic dietary therapy; epilepsy pregnancy registry, lactation, pregnancy outcomes

## Abstract

Ketogenic dietary therapies (KDTs) are increasingly used worldwide as a non‐pharmacological treatment for epilepsy, including among people of childbearing potential. In many cases, KDTs allow for the reduction of antiseizure medications (ASMs) and lower drug exposure during pregnancy. However, limited evidence on maternal tolerability, fetal safety, and lactation outcomes has led to reluctance among healthcare providers to recommend KDTs during pregnancy. To inform evidence‐based counseling, it is crucial to systematically collect data on maternal and offspring outcomes following KDT exposure. Over the past 25 years, international epilepsy pregnancy registries have provided robust data on the teratogenic risks of ASM exposure. Yet, no standardized dataset currently exists for documenting KDT exposure during pregnancy and lactation. The International League Against Epilepsy Ketogenic Dietary Treatments Task Force conducted a structured consensus process to develop the first international core dataset designed to capture maternal KDT exposure within existing pregnancy registries. This dataset provides a standardized, pragmatic framework for recording diet type, exposure duration, maternal‐fetal outcomes, and lactation parameters. By harmonizing data collection across registries, this initiative aims to transform isolated case reports into aggregated, high‐quality multicenter evidence, closing a critical knowledge gap. Incorporating these standardized questions into epilepsy pregnancy registries represents both a scientific necessity and a call to action to ensure safer, evidence‐based care for women with epilepsy and their children.

**Plain Language Summary:**

More people are using ketogenic diets as a treatment for epilepsy around the world, including during pregnancy. It is not yet known if these diets are safe for mothers and babies during pregnancy and breastfeeding. Collecting standardized information about pregnancy outcomes in existing registries will inform us about the safety and effectiveness of these diets. The authors, who are either members of the International League Against Epilepsy dietary task force or specifically chosen by them for their expertise, followed a consensus process to suggest the addition of key standardized questions to the existing international pregnancy outcome registries for epilepsy.

**Key points**

- The use of ketogenic dietary therapy among people of childbearing potential is increasing worldwide.
- Data on maternal and fetal tolerability and outcomes in pregnancy and lactation remain limited.
- Standardized registry data will generate the needed evidence on the safety and efficacy of the ketogenic dietary treatment.
- The authors propose a set of core questions to be included in international epilepsy pregnancy outcome registries.
- This consensus initiative is coordinated by The International League Against Epilepsy Ketogenic Dietary Treatment Task Force.

## 1 INTRODUCTION

Ketogenic dietary therapies (KDT) are increasingly used to treat drug resistant epilepsy^1^ and other neurological and psychiatric disorders including in people with epilepsy of childbearing potential (PWECP). Maternal diet during pregnancy is of vital importance to maternal health and to the immediate and long‐term health of the offspring. Epidemiological studies have shown that a poor maternal diet increases the life‐long susceptibility for chronic disease in offspring.^2^, ^3^ Some concerns regarding the potential teratogenicity of KDT are supported by preclinical findings.^4^, ^5^, ^6^, ^7^, ^8^ However, other studies suggest potential benefits of a maternal ketogenic diet to rodents and human offspring in particular conditions.^9^, ^10^, ^11^, ^12^, ^13^ In PWECP, KDT is most often adjunctive to antiseizure medications (ASMs). If seizures are well‐controlled on KDT, seizure control may be maintained on fewer ASMs or lower doses during pregnancy thereby reducing the risk of teratogenesis from these treatments.

The role of ketones in placental metabolism is emerging, and more research in humans is needed.^14^ Published cases of KDT exposure during pregnancy in PWECP are limited.^4^, ^10^, ^15^ One possible teratogenic effect noted in the offspring of one woman on KDT and ASM was helical rim deformities of the ear at birth,^16^ reported in 1:6000 live births in the general population.^17^ A recent survey of current practice revealed that many healthcare professionals are reluctant to advise PWECP to start or continue KDT during pregnancy due to a lack of knowledge and experience regarding tolerability and outcomes.^18^ However, enquiries about KDT use during pregnancy are increasing from PWECP and people with Glut1 deficiency^19^ syndrome, for whom KDT is the optimal treatment.

Potential teratogenicity is a significant concern when utilizing any treatments during pregnancy, and considerable progress has been achieved in establishing and maintaining pregnancy registries for PWECP to identify these risks. The largest independent registries are the International Registry of Antiepileptic Drugs in Pregnancy EURAP, the North American Antiepileptic Drug Pregnancy Registry (NAAPR), the UK and Ireland Epilepsy and Pregnancy Register, the Kerala Registry of Epilepsy and Pregnancy, the Raoul Wallenberg Australian Pregnancy Register of Antiepileptic Drugs and the West China Registry.^20^ NAAPR was established in 1997 to allow pregnant women treated with ASMs in the United States and Canada (https://www.aedpregnancyregistry.org) to self‐report any major congenital malformations in their offspring using an online survey.^21^, ^22^ In 1999, EURAP, a prospective observational study of pregnancies with antiseizure medications, was launched in Europe. It was organized as a consortium of independent research groups and later expanded to several other nations worldwide. Today, 47 countries in Europe, Oceania, Asia, Latin America, and Africa have participated in the study, and more than 30 000 pregnancies have been registered so far (https://eurapinternational.org). Thanks to systematic and thorough work by several pregnancy registries, it is clear that some ASMs increase the risk of birth defects, and the likelihood of these birth defects are well‐described.^23^ The 2022 multinational comparative monotherapy analysis (Europe, US, Australia) and the 2024 JAMA Neurology registry update reinforce the risk hierarchy: highest risk for valproate; elevated risk for older agents like phenobarbital; near‐background risk for lamotrigine/levetiracetam; and intermediate risk for carbamazepine with dose‐dependent effects.^23^, ^24^ The EURAP and UK and Ireland prospective registries and dose–response analyses corroborate these patterns.^25^, ^26^

It has taken 25 years to collect robust data on teratogenic risks associated with ASM exposure^23^, ^27^ and there is now a need to include outcome data on KDT exposure during pregnancy and lactation, within the international epilepsy pregnancy outcome registries, to accurately inform clinicians and PWECP of the relative risks. Currently, NAAPR includes one question asking whether the ketogenic diet is being used as an additional treatment. The UK and Ireland registry, EURAP, and the Australian registry do not include any questions regarding KDT. Given the small size of this patient cohort, it would be cost‐effective and important to include the same questions in each registry to enable the collation of high‐quality, validated data across registries. Implementation of a standardized dataset of questions into pregnancy registries will transform isolated case reports of KDT exposure into the summated multicenter evidence that clinicians and families urgently need. Herein, the Ketogenic Dietary Treatment Working Group of the International League Against Epilepsy proposes a core dataset of questions reached by expert consensus for inclusion in international epilepsy pregnancy registries.^20^

## 2 METHODS

The International League Against Epilepsy (ILAE) KDT Task Force, composed of global experts in KDT management for individuals with epilepsy, led and coordinated this consensus initiative. Within the task force, a dedicated working group of eight international experts with complementary expertise was formed, including both previous members of the KDT Task Force and three additional co‐opted members selected for their clinical experience and expertise in managing adults on KDTs: three adult clinical nutritionists with extensive experience in KDTs; two child neurologists with expertise in epilepsy, rare metabolic disorders, and KDTs; two adult neurologists specialized in epilepsy and KDTs; and one pediatrician with expertise in clinical nutrition and KDTs. A series of structured, virtual meetings was conducted following a consensus‐based expert group methodology similar to the Nominal Group Technique,^28^ with the aim of developing a core dataset of questions to capture the outcomes of maternal exposure to KDT for inclusion within international epilepsy pregnancy registries. Multiple iterative stages were employed:

1. *Idea Generation*: Initial suggestions were collected from working group participants in the form of questionnaires which generated draft lists of topics, focusing on maternal KDT exposure during pregnancy.
2. *Independent Rating and Prioritization of Proposed Items*: Each proposed question was first rated individually by participants with respect to priority for inclusion.
3. *Structured Group Discussion and Refinement*: Questions were subsequently discussed and refined in group sessions. Multiple iterative rounds of rating, refinement, and group discussion were employed to achieve 100% agreement among members.

Draft questions addressed key domains including the type of diet (classical ketogenic diet vs. modified Atkins diet or other variants) and correlated parameters (ketogenic ratio or carbohydrate intake, ketone monitoring), concomitant ASMs or supplementation use, maternal, perinatal, and lactation outcomes.

Integration of the proposed KD questions into the existing registry questions will also capture aspects of the timing of KDT exposure, aligned with the capture of the timing of ASM exposure in each registry.

As with ASM exposure, this allows differentiation between exposure limited to specific periods of gestation and exposure spanning the entire pregnancy.

Each item was assigned a priority level using a traffic‐light system (red = essential, amber = important, green = optional). This prioritization was performed recognizing that pregnancy registries have different levels of resources which may not be sufficient to incorporate all proposed questions.

The structured, iterative methodology^29^ ensured the inclusion of clinically meaningful and feasible variables for multinational epilepsy pregnancy registries, providing a standardized framework to inform future research on efficacy, safety, and offspring outcomes. The proposed dataset was circulated to the broader ILAE KDT Task Force for feedback and final approval.

The final dataset was structured to harmonize with existing registry data while addressing the current gap where KDT‐related information is either absent or inconsistently collected. To facilitate feasibility across diverse settings and reduce respondent burden, items already covered by existing registries (e.g., maternal age, congenital anomalies) were referenced rather than duplicated. Skip logic was applied so that diet‐specific details are only collected when relevant, and lactation questions are presented only when breastfeeding is reported.

## 3 RESULTS

The consensus process undertaken by the ILAE KDT Task Force resulted in the development of a core dataset of questions to capture maternal exposure to KDT within international epilepsy pregnancy registries. Full consensus was achieved among the ILAE KDT Task Force working group members for the final question set and prioritization. The domains and prioritization are summarized in Table 1.

**TABLE 1:** Proposed core dataset for maternal ketogenic diet therapy exposure in international epilepsy pregnancy registries.

Query requests	Answers
Classical ketogenic diet	Yes/No
If yes: ketogenic ratio	Free text
Modified Atkins diet	Yes/No
If yes: net carbs/day	Free text
Low glycemic index diet/treatment	Yes/No
If yes: net carbs/day	Free text
MCT diet or supplement	Yes/No
If yes: quantity/day MCT	Free text
Did the diet change before and/or during pregnancy?	Yes/No
If yes: please describe	Free text
Did you check ketones during pregnancy?	Yes/No
If yes	
Urine, blood, breath?	Choose one or more
Did ketones change during pregnancy?	Yes/No
If yes	
Did they increase, decrease, remain the same?	Choose one
Increase/decrease/same/increase and decrease at different times	
Did you start any new supplements when planning pregnancy?	Yes/No
If yes: please list with dosages	Free text
Did you start any new supplements once pregnant?	Yes/No
If yes: please list with dosages	Free text
Do you plan to breast feed?	Yes/No
(Post‐delivery) Are you breast feeding?	Yes/No
If not, please describe	Free text
Duration of breastfeeding	Months
Reason for the suspension	
Normal transition to weaning	Yes/No
Early termination due to maternal issues	Yes/No
If yes: please describe	Free text
Early termination due to baby issues	Yes/No
If yes: please describe	Free text
Did you check ketones during breast feeding?	Yes/No
If yes	
Urine, blood, breath?	Choose one or more
Did they increase, decrease, remain the same?	Choose one
Increase/decrease/same/increase and decrease at different times	
Color codes	
	Essential
	Important
	Optional

*Note*: Questions are prioritized according to perceived importance for determining risks and benefits of use of KDT in pregnancy: Red = essential, Amber = important, Green = optional. Directional changes in ketone levels refer to changes relative to baseline values (pregnancy or pre‐lactation), depending on the timing of KDT initiation.

Abbreviation: MCT, Medium Chain Triglycerides.

Whether on ketogenic diet (Y/N) or on low glycemic index treatment (Y/N) is already included in the NAAPR. It has been included in our suggested essential questions.

Red (essential)‐priority items focused on defining exposure to KDT, co‐exposures, and essential maternal and offspring outcomes. Questions determined specification of the diet variant (classical ketogenic diet, modified Atkins diet, low glycemic index treatment, or other), and quantitative measures such as ketogenic ratio or net carbohydrate intake, as well as whether diet type or parameters changed during pregnancy. Essential information on prescribed nutritional supplementation during preconception or pregnancy was also included, given their relevance to safety and efficacy. Red‐priority items additionally captured lactation status while on KDT.

Amber (important)‐priority items enriched the dataset by providing information on ketone monitoring during pregnancy, the sample type used for measurement (urine, blood, breath), and trends in ketone levels across pregnancy, which may be relevant for understanding dose–response relationships and metabolic adaptation.

Rather than mandating quantitative ketone thresholds, the dataset captures whether ketone levels increased, decreased, or remained stable over the course of pregnancy. Although the degree of ketosis may be biologically relevant for fetal and neonatal outcomes, quantitative ketone thresholds were not included in the core dataset. This decision reflects substantial variability in measurement methods, target ranges, and clinical interpretation across centers and countries, which may limit cross‐registry comparability.

Moreover, ketone levels represent a continuous variable that is subject to significant fluctuations over time due to multiple physiological and external factors, making it difficult to categorize levels reliably over a prolonged time course. Capturing monitoring practices and directional trends in ketone levels was therefore considered a pragmatic compromise that balances biological relevance with feasibility and cross‐registry comparability.

Green (optional)‐priority items included detailed breastfeeding outcomes such as intention to breastfeed, duration, weaning process, reasons for early termination, and changes in ketone levels during lactation.

## 4 DISCUSSION

This consensus represents the first structured attempt to define a core dataset for documenting maternal exposure to KDT during pregnancy with the ultimate goal of determining actual and comparative overall risks and benefits. While systematic data collection on ASM exposure has been established for over two decades through international pregnancy registries,^21^, ^22^, ^23^ KDT has, to date, received minimal attention as a potential contributor to maternal and fetal outcomes, despite increasing use among PWECP^18^ and in rare metabolic disorders where it remains the treatment of choice.^10^, ^30^ The proposed dataset fills a significant gap by providing a standardized framework that can be incorporated into existing registries with minimal additional burden. The dataset is intentionally pragmatic: red‐priority items capture essential exposures and outcomes that are critical for assessing both maternal safety and offspring health, while amber and green items offer additional insights for registries and centers with greater resources or research focus (Table 1 provides an overview of the domains and prioritization).

The importance of this effort is underscored by the current uncertainty surrounding the safety of KDT during pregnancy.^31^, ^32^ Preclinical studies have yielded conflicting results, with some suggesting potential teratogenicity and others indicating beneficial effects on neurodevelopment.^5^, ^8^, ^9^, ^12^, ^33^ Human data remains scarce and largely limited to case reports or small case series, making it impossible to draw firm conclusions about risks and benefits.^4^, ^14^, ^16^, ^34^, ^35^ Without systematic data collection, clinicians lack the evidence needed to provide clear guidance to people who may benefit or have benefited from KDT but are also planning a pregnancy.^36^, ^37^

This proposal builds on the success of pregnancy outcome registries in epilepsy, which have demonstrated that robust, multicenter collaboration over time can provide reliable estimates of teratogenic risks associated with ASMs.^38^ Achieving similar progress for KDT will require consistent integration of the proposed dataset into registry platforms, collaboration among international groups, and ongoing refinement as new evidence emerges, mirroring processes for ASMs. Importantly, engagement with registry leaders will be critical to align feasibility, ensure consistent implementation, and maximize the quality of the collected data.^39^

Without robust neonatal and infant data, the evaluation of KDT safety during pregnancy and lactation remains incomplete. Long‐term pediatric follow up will be essential to ensure that the registry captures the full spectrum of maternal‐child health outcomes.

## 5 LIMITATIONS

Some limitations of this work should be acknowledged.

First, as with any consensus‐based methodology, the proposed dataset reflects the clinical perspectives and experiences of the participating international experts, which primarily included neurologists and nutrition specialists. Patient and Public Involvement (PPI), including individuals with lived experience of KDTs during pregnancy or lactation, as well as perspectives from obstetrics and neonatology, were not directly incorporated into this process. While this expert‐driven approach was intentionally chosen to maximize feasibility and rapid integration into existing registries, future refinements of the dataset should incorporate PPI input to ensure that patient‐relevant outcomes, acceptability, and contextual factors are fully addressed. Such perspectives may also be integrated through the established review and governance processes of individual registries, which can account for local contextual and cultural variability.

Second, the proposed questions have not yet been empirically tested or validated within pregnancy registries. Their feasibility, completeness, and utility across different international registry infrastructures remain to be evaluated. However, the primary aim of this initiative is to provide a pragmatic, standardized framework that can be readily implemented. Importantly, established pregnancy registries typically operate within robust governance and review frameworks overseeing question content, case ascertainment, data completeness, and user feedback from both women with epilepsy and clinicians. These mechanisms provide an appropriate structure through which the inclusion, performance, and refinement of KDT‐related items can be evaluated over time, supporting informed, context‐specific adoption and iterative improvement based on real‐world experience.

Finally, variability in resources and data collection capacity across registries may influence the degree of adoption of the proposed questions. The prioritization system (essential, important, optional) was specifically designed to mitigate these challenges by allowing flexible implementation based on local feasibility, while ensuring some shared domains that permit data collation across registries.

## 6 CONCLUSIONS

The safety and impact of KDT on pregnancy outcomes and offspring health remain largely unknown. This consensus represents the first coordinated effort to define a core dataset for documenting maternal KDT exposure during pregnancy and lactation and offspring outcomes. Harmonization of this effort across existing epilepsy pregnancy registries will enable the collation of high‐quality data from a small but clinically important population. The proposal to collect this data is more than an academic exercise; it is a call to action. Without systematically collected evidence, clinicians cannot provide clear, evidence‐based counseling on pregnancy and breastfeeding risks to people who benefit from KDT. The epilepsy community now has both the opportunity and obligation to incorporate standardized questions on KDT exposure into existing international epilepsy pregnancy registries to ensure that they remain comprehensive and clinically meaningful. Doing so will empower clinicians to use evidence‐based medicine to ultimately support safer, more informed, and effective care for people with epilepsy on KDT during pregnancy and lactation.

## CONFLICT OF INTEREST STATEMENT

Dr. Kverneland has received speaker honoraria from Nutricia/Danone. Dr. De Giorgis has received research grants from Jazz Pharmaceuticals, speaker and consultancy fees from Nutricia Gmbh, Vitaflo, Dr. Schar Kanso, and has served as a consultant/advisor for Longboard Pharmaceuticals and Dr. Schar Kanso. Dr. Healy has received speaker honoraria from Nutricia/ Danone. Dr. McDonald has received grant support from the American Heart Association and the American Epilepsy Society. Dr. Armeno has received speaker and consultancy fees from Nutricia/Danone, Ajinomoto/Cambrooke, and Vitaflo. Dr. Cervenka is a consultant for Nutricia North America/Danone, Dr. Schär and Nestle Health Science, as well as a speaker for Nutricia North America/Danone, Nestle Health Science, The Neurology Center in Rockville, Maryland, and Dr. Schär. She receives royalties from Demos/Springer Publishing Company. Dr. Devlin has received honoraria for advisory boards, speaking, and training from Nutricia, Takeda, Zogenix, GW Pharma, Biocodex, and UCB. The remaining authors have no conflict of interest to disclose. We confirm that we have read the journal's position on issues involved in ethical publication and affirm that this report is consistent with those guidelines.

## Data Availability

Data sharing not applicable to this article as no datasets were generated or analysed during the current study.
